# The Place of Religiosity and Spirituality in Frankl’s Logotherapy: Distinguishing Salvific and Hygienic Objectives

**DOI:** 10.1007/s10943-023-01760-4

**Published:** 2023-02-07

**Authors:** Joaquín García-Alandete

**Affiliations:** Department of Personality, Evaluation and Psychological Treatments, Faculty of Psychology and Speech Therapy, University of València, Avda. Blasco Ibáñez, 21, 46010 València, Spain

**Keywords:** Viktor E. Frankl, Logotherapy, Religion, Spirituality, Criticism

## Abstract

The relationship between psychology and religion has been widely debated in the field of psychology from its foundation as an empirical science to the present day. One author who was interested in the relationship between psychology and religion, the place of the latter in human nature, and its role in psychotherapy was the Viennese neurologist, psychiatrist, and philosopher Viktor Emil Frankl (1905–1997), the founder of logotherapy. This paper presents Frankl’s main ideas about religion, the religious nature of the human being, and the relationship between religiosity, psychotherapy, and logotherapy, as well as a review of the main criticisms he has received in this regard. Frankl always defended the differences and limits between religion and psychotherapy, between the priestly cure of souls and the medical cure of souls, and between the salvific objective of religion and the hygienic objective of psychotherapy. In our opinion, critical authors have failed to appreciate Frankl’s efforts to expose this distinction.

## Introduction

The relationship between psychology and religion has been widely debated in the field of psychology from its foundation as an empirical science to the present day. This relationship has been considered in many different ways: as a neurotic phenomenon typical of people who were psychologically immature and/or ignorant of science and, ultimately, an illusion destined to disappear (Freud, 1907, 1913, 1927), as a non-scientific phenomenon and a tool for power and social control (Skinner, 1971), or as messages communicating peak experiences of the prophets or founders of the various religious traditions (Maslow, 1964).

These three authors cited above represent, as we know, the so-called three forces in psychology: psychoanalytic, behaviorist, and humanist, respectively. Evidently, a whole myriad of authors, some more relevant and others less so, have expressed their opinions about religion and its relationship with psychology, such as James (1902), Dewey (1934), Jung (1938), Allport (1950), and Fromm (1950), among others. We cannot present these opinions here, and so a few references about the relationships between psychology and religion will have to suffice for those interested (e.g., Jonte-Pace & Parsons, 2002; Malony, 2015; Nelson, 2009; Richards, 2011; Sykes, 1999, 2010).

One author who was interested in the relationship between psychology and religion, the place of the latter in human nature, and its role in psychotherapy was the Viennese neurologist, psychiatrist, and philosopher Viktor Emil Frankl (1905–1997), who founded logotherapy. This paper presents Frankl’s main ideas about religion, the religious nature of the human being, and the relationship between religiosity, psychotherapy, and logotherapy, as well as a review of the main criticisms he has received in this regard. Frankl survived the Holocaust and represented, along with Rudolf Allers (1883–1963) and Oswald Schwarz (1883–1949), the so-called «Third Viennese School of Psychotherapy» (Längle, 2012, 2015).

Frankl developed logotherapy from phenomenology and existentialism (Frankl, 1958, 1967a) as an alternative motivational theory on human beings and psychotherapy to both Freudian psychoanalysis and Adlerian individual psychology (Frankl, 1955). According to Frankl, the person is essentially motivated to search for meaning in life, which is personal and non-transferable, in his/her current circumstances. Likewise, logotherapy emphasizes that consciousness, freedom, and responsibility are the most important traits of human beings. Thus, meaning in life, will to meaning, and freedom of will are the essential issues of logotherapy, in opposition to pan-determinism, homeostasis theory, and reductionism (e.g., Frankl, 1965).

Although Frankl has been internationally recognized as an influential thinker, logotherapy has been the topic of controversy and criticism for decades and up to the present day (e.g., Arnold & Gasson, 1954; Biller et al., 2002; Bulka, 1978; Frankl, 1979; Maslow, 1966; May, 1961, 1978; May et al., 1958; O’Connell, 1972; Pytell, 2006, 2015, 2016). One of the polemic issues had to do with the role of religion in logotherapy, in several senses: (1) the place of spirituality and religion in human nature according to logotherapy, (2) the place of religion in psychotherapy in general and in logotherapy in particular, and (3) psychotherapy in general and logotherapy in particular as a medical cure for the soul (e.g., Bulka, 1971, 1975; Crumbaugh, 1979; Dickson, 1975; Fabry, 1975; Grollman, 1965; Okan & Ekşi, 2017; Palmer, 1987; Péter, 2008; Sellés, 2016; Shea, 1975; Sykes, 1999, 2010; Weisskopf-Joelson, 1975; Yalom, 1980; Yildirim, 2018).

## Transcendence in Frankl’s Logotherapy: The «Spiritual-as-noetic» and the «Spiritual-as-religious»

Frankl deals with the analysis of existence, which is understood as a personal itinerary in the search for meaning in life, whose achievement would result in maturity and self-realization (Frankl, 1962a). Franklian existential analysis constitutes a set of reflections on the existence of the human being, a set of meta-clinical assumptions of logotherapy. All psychotherapy is based implicitly or explicitly on both a certain theory about human beings and a world view, which constitute its aprioristic horizons.

Frankl distinguishes three dimensions in the person’s structure: body, psyche, and noos or spirit. The first two together form the psychosomatic structure of the individual, and the third is related to the aspects that transcend the psychosomatic. According to Frankl, whereas the bodily is inherited, the psychic is educated, and the spiritual is realized (in other words, it matures). Likewise, the body is the condition of expressibility of what the psychic realizes as a requirement of the spiritual. The psychophysical organism is the person’s executive and expressive dimension.

However, the person does not result from the mere addition of these three dimensions, but rather he/she is a single unity (Frankl, 1969). As Sykes (2010) noted regarding the spiritual dimension in logotherapy:

The spiritual dimension represented the core of the human psyche. It embodied wholeness and the union of body, mind, and spirit. Each dimension, beginning with the somatic, permeated the next to depict ever-expanding and inclusive levels of human consciousness. The spiritual dimension represented the location of and source for what Frankl identified as truly human phenomena. (54)

The corporeal is determined by biological inheritance, in such a way that man finds him/herself in a body not chosen by him/her or his progenitors. This corporeality is related to the way of being, to the way of expressing being, and not to existence itself. Man, then, *has* a body and a psyche, but *is* spirit, so to say. Existentiality is essentially united with spirituality in the case of the human being.

Man can place him/herself above his/her factual, psychophysical conditionality (factual conditionality *versus* facultative conditionality). The anthropological datum of freedom confronts the psychophysical conditions, where the conditions and circumstances do nothing but place limits on the real and genuine faculty of deciding on the part of the person. In other words, *bios* is not a cause of *logos*, but a mere condition, and cause is not equal to conditions. In this regard, conditioned is not equal to caused, as facticity is not equal to existentiality, and the psychophysical (factual dimension of being) is not equal to the spiritual or spiritual person (existential dimension of being).

In Frankl’s logotherapy, two distinct meanings can be distinguished in relation to what he considers “spiritual,” understood in turn as transcendent: (1) spiritual as noetic and (2) spiritual as religious. The noetic dimension is related to the personal will to meaning, and in this case, logotherapy is a therapy of logos, that is, of meaning as human spiritual longing (Frankl, 1962b):

To avoid confusion arising from the fact that the term “spiritual” usually has a religious connotation in English, I prefer to speak of no&ie, in contrast to psychic phenomena, and the noo1ogical, in contrast to the psychological dimension. The noo1ogical dimension is to be defined as the dimension where specifically human phenomena are located. (94)

In this regard, Frankl stated that “the will-to-meaning is the subjective side of a spiritual reality in which the meaning is the objective side; at least it is objective insofar as the will is concerned with “finding” meaning and not at all with “giving” it” (Frankl, 1959, 163). Likewise, Frankl pointed out (1959):

One characteristic of human existence is its transcendence. That is, man transcends his environment toward the world (and toward a higher world); but more than this, he also transcends his being toward an ought. Whenever man transcends himself in such a manner, he rises above the level of the somatic and the psychic and enters the realm of the genuinely human. This realm is constituted by a new dimension, the noetic; it is the dimension of spirit. Neither the somatic nor the psychic alone constitutes the genuinely human; rather, they represent only two sides of the human being. Thus, there can be absolutely no talk of a parallelism in the sense of dualism, nor of an identity in the sense of monism. Nevertheless, in spite of all the ontological variations of the somatic, psychic, and noetic, the anthropological unity and wholeness of a human being are preserved and saved as soon as we turn from an analysis of existence to what I call a dimensional ontology. (159)

## Logotherapy and Religion

In 1948, Frankl published *Der Unbewusste Gott. Psychoterapie und Theologie*, which was the result of a lecture he had previously given to a group of Viennese intellectuals, and with which he obtained his doctorate in philosophy. In 1975, this book was published for the first time in English, under the title *The Unconscious God. Psychotherapy and Theology*.

In 1997, Frankl’s book *Man’s Search for Ultimate Meaning* was published. It was the result of another conference given at the national meeting of the American Psychological Association held in 1985 in Dallas when the Oskar Pfister Prize was awarded to Frankl. This book is a revision and expansion of *The Unconscious God*.

In 2005, *Gottsuche und Sinnfrage: Ein Gespräch* [*The Search for God and the Meaning of Life. Dialogue between a Theologian and a Psychologist*] was published. It was co-written with the Jewish theologian and Israeli diplomat Pinchas Lapide (1922–1997) and was an edition of a conversation they had about religion (Frankl & Lapide, 2005). These books included a reedition of many articles that Frankl had published during the previous decades or that contained his ideas about religion and its relationships with psychology and psychotherapy (e.g., Frankl, 1954, 1959).

## Spiritual Unconscious, Transcendence of the Consciousness, Unconscious Religiosity

Frankl’s existential analysis deals with religiosity in order to avoid incurring in the absolutization of the human person, turning him/her into God, once his bio-psycho-social conditionings have been relativized. One of Frankl’s ten theses on the person stresses man’s necessary openness to transcendence for his ontological self-understanding (e.g., Frankl, 1969). In other words, we could say that man is constitutively religious, relative, and respective of transcendence.

Frankl had every reason to stress the relative, not absolute, freedom of man: the human person is a mere creature; he/she is not God. We could say that Frankl’s existential analysis is interested in “the absolute” (God), in order for “the relative” (human being) to remain such, in relation to the ontological condition of man’s responsibility. Furthermore, existential analysis is interested in the absolute for psychotherapeutic reasons, rather of a prophylactic order, given that the religious experience provides the person with a “refuge,” with no other attitude being more beneficial (in a psycho-hygienic sense) than the religious one in facing extreme situations.

In addition, although existential analysis cannot answer questions about the ultimacy of human existence, at least those related to religiosity, it can guide the person toward religious questions because its final objective coincides with that which leads the person to religion. It is not possible to speak of man without referring to transcendence, and any effort to understand man without reference to transcendence is doomed to failure. The opposite is anthropocentrism and nihilism, which derive in axiological relativism, given that value judgments can only be issued if they are anchored in a referent of absolute value. This absolute value refers to a “super-person.”

The relative, the conditioned, does not exclude the absolute, the unconditioned. On the contrary, the conditioned presupposes the unconditioned. Therefore, God is, as an absolute value, the necessary referent in the axiological. When one forgets or ignores this absolute axiological referent (that is, God), he/she deifies and absolutizes what is relative and enters into what Frankl called the “Faustian man.”

Frankl’s central theses in relation to the religious nature of human beings are the following. First, there is a religious meaning anchored in the personal unconscious that is related to an ultimate meaning or meta-sense, which can either be experienced and lived consciously or remain hidden or ignored but emerge suddenly and unexpectedly in the presence of the most varied events. In this regard, man is a being immanently inclined toward transcendence. Second, consciousness is a transcendent phenomenon, not a mere “immanent psychological fact,” and it can only be fully understood when it is referred to a transcendent origin, which goes beyond the framework of psychological immanence. Third, there is an unconscious religiosity. Below, we will develop the core aspects of these three theses.

### Spiritual Unconscious

The question of the unconscious is central to Frankl’s thought. In his review of the “boundaries of the unconscious,” he warns that it is not reduced to the impulsive, as maintained by Freudian psychoanalysis, because it also has a spiritual component (spiritual unconscious). Just as there is an unconscious impulsivity (which Frankl did not deny; he simply objected to it being considered in exclusivist and reductionist terms), there is an unconscious spirituality (which does not mean ignoring the conscious one). Moreover, the character of necessity of the spiritual lies precisely in its unconscious nature.

This requires identifying the boundaries that separate unconscious religiosity from conscious religiosity. These boundaries are blurred because, on the one hand, the conscious can be repressed, and on the other, the repressed can become conscious, so that one can speak of a “relativization of the state of consciousness.” Thus, consciousness cannot be the ultimate reference. Assuming what other existentialist authors, such as Jaspers, Heidegger, and Biswanger, stated about man as a “being-that-always-decides,” as a self-determining being, Frankl understands that man can be himself that the person maintains him/herself as him/herself, even in the realm of the unconscious.

The spiritual unconscious is understood by Frankl as an unconscious tendency toward God, not instinctual but intentional. It is the “ignored presence of God” or the “unconscious God,” an expression Frankl uses to mean that, at times, God remains hidden in the unconscious, that is, that one’s relationship with Him is repressed and, to that extent, unknown to oneself. This relationship is veiled by ignorance of a God who remains hidden.

Despite their unconscious nature, spirituality and religiosity cannot be reduced to a simple “product” of the *Id*: “man is often more religious than he himself suspects. But we must not make the mistake of looking upon religion as something emerging from the realm of the id, thus tracing it back again to instinctual drives” (Frankl, 1954, 42).

One of the ways the spiritual unconscious expresses itself is through dreams, and through their existential analysis, Frankl discovers the phenomenon he calls “religious modesty”; in other words, something that is intimate and should be kept away from the gaze of strangers becomes the object of public exposure. Thus, modesty fulfils a protective function, preventing something that is sacred from being profaned.

Religiosity’s character as an absolute object comes to mean that it is something essential and truly intimate, reaching its highest level of authenticity in the intimacy of the depths of the person. In the same way that modesty keeps religiosity away from strange, profaning glances, another psychic dynamic, repression, can hide it from the conscious self, turning religiosity into something that, despite existing, is nevertheless not consciously experienced by the person himself.

### Transcendence of Consciousness

The transcendent nature of consciousness refers to the freedom of the person in a double sense: “freedom-from” the driven and instinctual, and “freedom-to” responsibly decide the course of one’s actions. Consciousness is different from the self. It has an “extrahuman” character. It is a phenomenon that transcends the individual in his/her psychological facticity and through which it is possible to understand him/herself. Consciousness is more than a mere “immanent psychological fact,” and it can only be fully understood when it is conceived as transcendently originated and rooted.

Taking the above into account, religious unbelief consists in the ignorance of the transcendent character of consciousness, which is considered in its mere immanence, that is, in its mere psychological facticity. The unbelieving person simply leaves in suspense the question about the origin and transcendent referent of consciousness itself. Consciousness is a penultimate reality and not an ultimate one (insofar as it refers to someone superior to itself), and the irreligious man is one who suspends or stops his search for meaning at the limits of consciousness, as psychological facticity.

By referring to transcendence (authentic ultimacy), consciousness is ontically irreducible, the problem associated with it being of an ontological and not psychological order. In contrast to the Freudian pretensions to reduce this ontological condition of the consciousness (exemplified by the “religion of the father,” with which Freud denies the transcendence of the consciousness and its relationship with the transcendent; as is known, the father complex is the Oedipus complex, the origin according to Freud of religion, morality, art, and society), Frankl maintains that, in reality, divinity is not an “image of the father,” but rather paternity is an image of it. In the ontogenetic order, the first is the biological father, but in the ontological order, the first is God. With these ideas, Frankl lashes out against the Freudian psychologistic reductionism of consciousness and the experiences associated with it.

### Unconscious Religiosity

Frankl’s reflections on unconscious religiosity follow a series of stages. The starting point is the anthropological data on both consciousness and responsibility, which are summed up in the fact that the person is aware of his/her responsibility.

The second stage is that of entering into the realm of unconscious spirituality. Recognizing the spiritual alongside the psychic, logotherapy learns to see and teaches to see the spiritual dimension within the unconscious. An unconscious responsibility is postulated together with the conscious responsibility, and, with the recognition of this unconscious spirituality, existential analysis avoids two aporias: on the one hand, the psychoanalytic elloification of the unconscious and, on the other, rationalist reductionism, where the person is conceived exclusively on the basis of reason, both theoretical and practical.

The third stage is the discovery of unconscious religiosity as an unconscious state of a transcendent relationship with God, in which the transcendent you becomes visible behind the immanent self. We have dealt with this above on the subject of the transcendence of the spiritual unconscious and the «unconscious God». According to Frankl, there are three tendencies that must be avoided in order not to distort the concept of the (unconscious) relationship with God: pantheism, occultism, and independence from the ego.

In relation to this, as opposed to Jungian archetypal postulates, Frankl considers that the origin of religiosity does not lie in the collective unconscious, but rather it depends on a profound personal decision. In Frankl’s opinion, Jung’s mistake consists of having considered religiosity from the psychophysical point of view of the unconscious, and not from the existential, spiritual point of view. Religiosity emanates from the deepest part of the person. This does not mean, however, that Frankl considers religiosity to be something innate and biologically determined, precisely because of its spiritual and not psychophysical character.

Moreover, the constitutive religiosity of man would be channeled through the existing religious schemes in the sociocultural context to which one belongs, and not by virtue of a supposed inheritance of a collective unconscious (Frankl, 1954):

Once I was asked after one of my lectures whether I did not admit that there were such things as religious archetypes, since it was remarkable that all primitive peoples ultimately reached an identical concept of God, and this could after all only be explained with the help of a God-archetype. I asked my questioner whether there was such a thing as a Four-archetype. He did not understand immediately, and so I said: “Look here, all people discover independently that two and two make four-perhaps we do not need an archetype for an explanation-perhaps two and two really do make four. And perhaps we do not need a divine archetype to explain human religion either-perhaps God really does exist”. (42)

For Frankl, the first early, infantile, religious experiences are of the utmost importance, and he notes that the religious outbreaks that are sometimes experienced by those who have repressed their unconscious religiosity are linked to them. But in no case does Frankl refer to an archetypal type of religiosity (Jung), which would be nothing more than «archaizing mythology».

When religiosity is repressed (nevertheless always present), existential analysis proceeds to its actualization. The repression of religiosity, which would be a deficiency, supposes a disturbed relationship with transcendence. However, it is possible for the repressed religiosity to emerge (the «restlessness of the heart»), expressing itself neurotically, which would be a «wretchedly repressed» religiosity, psychically disturbed, a reflection of the deficit of transcendence.

Frankl warns that God should not be considered a thing, reified, but rather experienced as a person, as a You with whom one enters into an intimate dialogue. Nevertheless, from the existential analysis of religious experience, one cannot demand logical proof of its existence. On a strictly personal level, Frankl considers such proof to be blasphemous because proof can only be found of «the ontic», of what lies within the realm of the natural, but not of what lies beyond that realm.

Metaphysical interpretations are indications but not proofs, and access to God, in any case, has an ontological nature, just as a possible valid demonstration of his existence must be phenomenological (in this regard, the person can close him/herself to the attempt at phenomenological demonstration by repressing his/her metaphysical need).

The relationship with God is always an interior vis-a-vis, a you-to-you dialogue, because God is the original You. It is a cordial rather than intellectual dialogue as befits the relationship with God. If we try to grasp God conceptually, we annihilate him (we make him nothing), and if we recognize and admit him in his lack of apprehensibility and ineffability, then we recognize him in his transcendence and absolute value. It is in prayer that the Thou who is God is saved, presentizing, concretizing, and personalizing Him. Prayer, on the other hand, does not necessarily require words.

Moreover, silent prayer can be the most religious of prayers. Prayer crystallizes in the symbol, which allows access to transcendence: not with the symbol, but in the symbol. Symbolism is a human need, and its reason is not rational utility, but rather the satisfaction of the reasons of the heart (Frankl, 1975).

In addition, in relation to the question of the essence of God, the approach has to be dialectical and involves the acceptance of a paradox: God is, at the same time, absolutely transcendent and absolutely immanent, absolutely distant and absolutely close. His transcendence is related to his unthinkability and ineffability, subtracting the option of believing in Him and loving Him, which is always a personal decision (Frankl, 1975).

### Beyond Temporality

According to Frankl, although man cannot give a rational answer to all his concerns, especially the question of whether there is an ultimate meaning to all things, he can give them an existential answer, an answer that is beyond the rational and points to faith:

[...] the finitude of the human spirit makes (...) that man can only have access to a particular meaning: the sense of totality exceeds human capacity, and only a limited concept such as “supermeaning” is offered as an answer to the “longing for meaning”. But at this point, knowledge is transmuted into faith; and it can be shown by way of casuistry that faith in supermeaning is most obvious once the effort of thought, the “work of the concept”, prepares the way for it.^1^ (Frankl, 2000, 45)

Man’s existence, in its earthly, temporal dimension, has to be open and projected toward a suprasense, which is realized in a supra-time (in eternity) in order to reach the fullness of its potential for meaning. Only in transcendence is it possible to find absolutely genuine and fulfilling meaning. As an original and ultimate instance, it is not the person who gives the ultimate meaning to his existence (nor, obviously, existence itself, with life being the fundamental condition of the search for meaning), but God. In this connection, he wonders whether the world of man is a final station without anything beyond it, or whether, on the contrary, there might be another world beyond the human one that refers to a supersense.

We could highlight something: God is the Transcendent Point, so to speak, toward which man can orient him/herself, intensively projecting all the moments of existence. If this is true, personal existence can flow continuously, gaining in transcendence; it can flow through time, becoming eternal. This existential tension toward fullness, which is not scientifically demonstrable but can be experienced phenomenologically as authentic, is experienced by the person as a deep longing, as a thirst for infinity, which only God can fulfil, both during the events of earthly life and after it, because the life of man is not conceivable if we ignore its radical root in Transcendence from the beginning and for all eternity.

However, it does not seem to be clear what Frankl means by “ultimate meaning.” Is it just a philosophical idea or is it the religious dimension of meaning? Sometimes he refers to it in a religious sense, whereas other times he refers to it just in a philosophical sense. For example, in his autobiography, Frankl (2000) wrote:

The other basic idea I developed in my early years maintains that ultimate meaning is, and must remain, beyond our comprehension. There exists something I have called “suprameaning”, *but not in the sense of something supernatural*. In this, we can only believe. In this, we must believe. Even if only unconsciously, essentially, we all do believe in it.

[…] whatever we have to go through, life must have ultimate meaning, a suprameaning. This suprameaning we cannot comprehend; we can only have faith in it. (56).

However, people do not have faith in philosophical ideas, but they have faith in God. Therefore, if the suprameaning is not God, what is it? What is that suprameaning and how do we experience it? It is not clear what the nature of this not-supernatural-supermeaning is. Frankl does not offer satisfactory answers to these questions. For the non-believer, it would seem that such a suprameaning is nothing more than a merely aesthetic or diffuse spiritual experience.

To this, we must add that the reference to transcendence, to God and to super-meaning could be, in Frankl’s case, essentially linked to his personal experiences of suffering (Pytell (2015):

Frankl’s positing of a spiritual dimension seemed to suggest he believed in the reality of a world that transcended time and space. Clearly this was the thrust of his play, his “communication” with his wife in his Holocaust testimony, and his belief in God. Arguably this flight from material reality was deeply refl ective of how he responded to his traumatic wartime experience. After those experiences, Frankl posited a will—to meaning—that could only come from a transcendent source. (139)

## Religion in the Psychotherapeutic Relationship: The “Medical Cure of the Soul”

In several works, Frankl alludes to the question of the substitution of the priest by the therapist in order to deal with questions that are not truly clinical, but moral and existential (e.g., Frankl, 1954):

A well-known psychiatrist once said that Western Humanity had turned away from the priest to the doctor. Another psychiatrist complained that nowadays so many patients approached the medical man with problems which should really be put before the priest; but when one tried to send them to a priest, they would not go. Actually, we find that patients repeatedly come to us with such problems as the meaning of their existence. It is by no means true, however, that we doctors attempt to carry philosophy over into medicine, though this is often said of us; it is the patients themselves who bring us philosophical problems—the problems of their own concept of life. (37)

In this quote, Frankl offers his position on the question of the relationship between religion and psychotherapeutic practice. In his opinion, the psychotherapist may reduce the existential problem to one of a psychological nature (that is, the psychotherapist psychologizes an existential or spiritual problem). However, even if there is no professional interest in the patient’s religiosity, when it appears in the context of the psychotherapeutic relationship, spontaneously on the part of the patient (especially when it is a matter of religiosity that has been repressed, it is useless to try to force it to emerge), an absolute tolerance must be shown toward it. This would be a sign of respect for the patient as a being who decides, that is, as a conscious, free, and responsible being, where the task of existential analysis is none other than to ensure that he/she comes to assume it existentially.

Furthermore, Frankl recognizes the spiritual dimension in the human person, “the very one which makes a being human” (Frankl, 1954, 37), together with both the somatic and mental. A psychologizing conception of the human being does not recognize the spiritual dimension of his nature and, therefore, denies the reality and importance of religiosity.

Frankl’s conception of religion has nothing to do with confessionalism (which he called “confessional narrowness” and “religious myopia”), where God is considered a being who, like an accountant determined to fatten his client portfolio, is exclusively interested in having more and more believers. Nor does he believe that God demands that we believe in him, because faith cannot be forced, just as one cannot be forced to love someone in an authentic way. In this regard, authentic religiosity is a free act of the will, a personal adherence, and an intentional act.

In relation to the hackneyed question of the modern substitution of the priest by the psychotherapist in our days, on the one hand, Frankl maintains that nothing could be further from the medical cure of souls than the pretension of substituting the priestly cure of souls, and that using religion as a psychotherapeutic means would lead to its degradation, turning it into a kind of sufficiently effective drug.

On the other hand, the medical cure of souls would be a complement to psychotherapy, addressed not so much to the believer (who already, in a certain way, can feel protected in his religiosity) as to the unbeliever, given that he lacks a transcendent referent on which to rest the burden of his existential sufferings. Likewise, it would be a psychotherapeutic requirement, especially with patients who are faced with an unchangeable destiny, for whom it is no longer a question of curing, but of consoling, of procuring spiritual relief, although this does not and should not mean that it is a substitute for priestly pastoral care (Frankl, 1954; Cfr. Graber, 2016; Laracy, 2019).

The consoling action of the medical cure of souls (that is, the competence of the psychotherapist not only as such, but also as a human being) implies, in cases where the patient is facing an irreversible situation that is causing significant suffering, offering “last aid,” which would consist of making him/her see that it is quite possible to find meaning in spite of everything. Finally, this dimension can even become a requirement for the psychotherapist, inasmuch as he cannot deny consolation to the patient who needs it. This consoling dimension is a medical responsibility beyond the assistance provided by pastoral care (Frankl, 1967b).

Now, it is possible that psychotherapeutic action may result in the health of the soul, although as an effect and not by intention, just as it is possible that, also as an effect and not by intention, religion may result in psychic health (e.g., confession can bring relief and unburdening of the conscience). But because religion is situated on a higher plane than psychotherapy, the irruption of the latter into the former is in the field of faith, and not the field of knowledge:

Logotherapy is no substitute for psychotherapy, but its complement; but least of all does medical spiritual care aspire to be a substitute for the proper care of souls; that is practiced by the priest. Now, what is the relation between the medical and the priestly care of souls? What is the relation between psychotherapy and religious care? In my view, the answer is simple: the goal of psychotherapy is to heal the soul, to make it healthy; the aim of religion is something essentially different-to save the soul. So much for the different aims of psychotherapy and religious care of souls. But if, instead of asking what is being aimed at we try to see the result, that is, the unintended side-effect, we will find that the side-effect of religious care of souls is an eminently psycho-hygienic one. This is due to the fact that religion provides man with a spiritual anchor and a feeling of security that he cannot find anywhere else. But to our surprise, psychotherapy can produce an analogous unintended side-effect because, although the psychotherapist is not concerned with, must not be concerned with, helping his patient to achieve a capacity for faith beyond the restitution of his capacity to work, enjoy, and suffer, in certain felicitous cases, the patient regains his capacity for faith, even though during his psychotherapeutic treatment neither he nor his doctor had aimed at this end. (Frankl, 1954, 39)

Psychotherapy cannot go beyond its own limits, not even existential analytical logotherapy (the medical care of souls), illegitimately incurring in the field of priestly cure. The religious response to the final meaning of existence is deeper than what existential analysis can offer. Nevertheless, existential analysis can be a way for the religious problematic to become conscious to the person, so that it can be recovered from the unconscious and existentially integrated.

Although religion is, as has been pointed out, an “object” of psychotherapy, the latter is interested in “meaning” because of the human existential tension (Cfr. Längle, 2016). There is an ultimate meaning beyond which man can only remain reverently silent, and this faith in meaning is for Frankl a transcendental category, present even in the most convinced atheists, a meaning that emerges from the depths of existence. And when the question of faith is considered not so much in relation to belief in God, but in relation to meaning, psychotherapy must deal with it. As Längle stated in accordance with Frankl’s ideas, “since existential analysis works explicitly and practically with the personal-spiritual dimension of the patient, it is appropriate to deal with a patient’s implicit relationship to spirituality” (Längle, 2016, 38; Cfr. Reitinger, 2017).

Moreover, logotherapy can deal not only with the question of meaning, but also with the question of “meta-meaning” or “supra-meaning,” to which religious faith ultimately refers. The question of “supra-meaning” refers to the suprarational, to that which remains beyond the limits of pure rationality, to man’s incapacity to know “the absolute,” the “absolute meaning.” His possibilities are limited to understanding personal and concrete meaning, hic et nunc, ad personam et ad situationem. The question of whether life, in its unfathomable totality, has meaning escapes the human capacity for rational understanding (Frankl, 2003):

The answer to the question of absolute meaning is beyond the possibilities of the human being. (...) the question of meaning fails as soon as it is applied to totality. The totality, in effect, is unreachable, and for that reason, its meaning necessarily exceeds our capacity for comprehension. The meaning of the whole is ineffable, unaffordable, even in the line of a limited concept, so that it is possible to affirm that the whole lacks meaning because it has a supersense. But “supermeaning” has nothing to do with the “suprasensible” and signifies “suprarational” here. (246)^2^

In contrast, the “consolation of souls,” which would also be a psychotherapeutic task when necessary, would in no case involve the prescription of a meaning, which would imply its moralization: meaning cannot be imposed or prescribed, but discovering it is a personal and non-transferable task (however, a certain moral obligation is implied because the choice of meaning by the patient must be personal and responsible) (e.g., Frankl, 1954).

## A Kind of Secular Religion?

It is not a secret that Frankl was a devote Jew, and logotherapy is influenced by his religious beliefs. Frankl’s writings are “literally interlaced with quotations from authoritative Jewish sources. His works breathe the very spirit of Judaism” (Grollman, 1965, 24). This is probably why Frankl’s writings seem to be more than a psychological theory of human motivation and a psychotherapy, to the point that some authors have stated that logotherapy is not a psychotherapy in a traditional sense, but a kind of philosophy of life (e.g., Johnson, 1968; Leontiev, 2016; Weisskopf-Joelson, 1975). They have identified essential similarities between logotherapy and what could be called “religious existentialism,” such as Paul Tillich’s theological works (e.g., Sykes, 1999), as well as the main religious traditions, that is, Judaism and Christianity (Cfr. Bulka, 1971; Grollman, 1964; Grossman, 1969; Tweedie, 1972), and Hinduism, Buddhism, and Taoism (e.g., Fabry, 1975).

Such similarities might be explained by the fact that logotherapy expounds, as existentialist philosophy and theology and the great religious traditions do, what would become fundamental aspects of human nature from an ethical and spiritual point of view: conscience, freedom and responsibility, search for meaning in life, and openness to transcendence (Cfr. Okan & Ekşi, 2017). However, for Frankl, psychotherapy and religion are different realms, although religion can have a positive effect on mental health, and, in turn, psychotherapy can open the door to religiosity for the patient:

The goal of psychotherapy, of psychiatry, and, quite generally, of medicine is health. The goal of religion, however, is something essentially different: salvation. So much for the difference in goals. The results achieved, however, are another matter. Although religion may not aim at mental health, it might result in it. Psychotherapy, in turn, often results in an analogous product. (Frankl, 1967b, pp. 45-46)

Despite this differentiation by Frankl between psychotherapy and religion, Sykes (1999) linked logotherapy to religion through the essential link between the «will to meaning» and the «ultimate meaning», which Frankl established in his writings:

Although Frankl never explicitly links the will to meaning to an ultimate meaning, his definition of religion implies that the meanings we discover in our lives are partial experiences of an ultimate meaning. If the spiritual dimension; (a) represents an unconscious yet unified dimension of human experience and endeavour, and (b) mediates between the “will to meaning” and an ultimate meaning, then the primary motivation within the human psyche would be an inherent religious motivation that seeks an ultimate concern. (74)

Moreover, in his review of Frankl’s book *Psychotherapy and Existentialism* (Frankl, 1967b), Williams (1968) stated:

He [Frankl] says that there is an essential difference between the aims of psychotherapy and religion. The goal of psychotherapy is health, and that of religion “is essentially different: it is salvation.” (pp. 32-33). But Frankl turns completely around and says that doubting whether life has a meaning is “a spiritual distress rather than a mental disease.” Here logotherapy becomes an assignment “to all the counselling professions.” (p. 67). In other words, logotherapy is here concerned with precisely the same problem as is religious faith. (289)

Yalom (1980) went further, affirming that logotherapy is a fundamentally religious theory of meaning in life:

[…] many scholars find Frankl's method offensive. His arguments are often appeals to emotion; he persuades, makes ex cathedra proclamations, and is often repetitive and strident. Furthermore, although he claims to present a secular approach to meaning (he states that as a physician who has taken the oath of Hippocrates, he is obliged to develop treatment methods that apply to all patients, atheists and devout alike), it is clear that Frankl's approach to meaning is fundamentally religious. (442)

Certainly, Frankl himself considers that logotherapy has its sources and its objectives beyond the purely psychological (Frankl, 1975, 1997). As he stated, logotherapy (Frankl, 1953):

[…] is a therapy which derives from spiritual sources and aims toward a spiritual goal. (…) Logotherapy proceeds from the spiritual because it not only presupposes Logos as an objective, sensorily-oriented, and value-directed concept, but it also applies it in the psychotherapeutic procedure.

On the other hand, logotherapy is directed toward a spiritual aim when, in the form of an Existential Analysis, it makes man reflect upon himself as a “spiritual subject”, when it points out to him his “subjective spirituality”, in short, his “existence”.

[…] the so-called mental (spiritual) diseases are not even diseases of the soul; they are not psychoses but somatoses, and as such they cannot ever become diseases of the mind. Where spiritual values are involved, there can be no question of illness; the categories “healthy vs. sick” cannot be used here. When speaking of the spirit, we cannot refer to pathology in any form; there is no nosological category involved, but only a noologic one, namely, “true vs. false”. (10-11)

For Frankl, religion stands as the ultimate and, therefore, most important human motivation. However, as noted above in relation to the differentiation between the somatic, the psychic and the noetic or spiritual, he states that the conception of spiritual and transcendence in logotherapy is not necessarily a religious one. Logotherapy “is such a secular science; that is its strength. Religion, or the spiritual sphere, is the proper concern of man in his search for ultimate meaning. Therein, the difference” (Costello, 2015, 6). However, one cannot deny the «religious vein» that runs through logotherapy from beginning to end, and one cannot ignore that eminent authors have questioned Frankl’s position on the relationship between religion and psychotherapy, whose frontiers may not have been well defined by the founder of logotherapy.

## Criticisms of Logotherapy as a “Religion”

Frankl (1975) relates that, on an occasion when he entered the room where his assistant Kurt Kocourek was conducting group therapy, a patient was lamenting the loss of her disabled son, to whose care she had devoted her whole life. Frankl presented the group with the example of a chimpanzee being used experimentally to find a vaccine against poliomyelitis, which ignores the sense of suffering that the experiment causes the animal.

Similarly, Frankl raises the possibility that there is a world beyond the present and a higher one, in which the ultimate meaning of suffering, an absolutely transcendent super-sense, could be found (Frankl, 1975). This means the introduction of a religious or at least a metaphysical element into therapy, which can be a therapeutically valuable resource when it is the patient him/herself who raises it in psychotherapy. Now, is it legitimate to raise such a possibility if the therapist does not believe in it and in its value, not merely therapeutic, but even human?

Rollo May (1961) severely criticized logotherapy, qualifying it as authoritarian, which generated a heated debate with Frankl (1975) and in which Bulka (1978) intervened in defense of Frankl. With regard to the question dealt with in the present work, Bulka (1978) deals very succinctly with the question of the “philosophy of logotherapy,” referring to a very clarifying text by Frankl (1975) in *The Unconscious God*:

Medical ministry operates along a great divide -the dividing line between medicine and religion. Anyone who walks along the frontier between two countries must remember that he is under surveillance from two sides. Medical ministry must therefore expect wary glances; it must take them into the bargain. (230)

According to Bulka in his response to May criticism to logotherapy, its philosophy “is explicit and its clinical application is clear and precise” (Bulka, 1978, 53). Our position on this matter coincides with Bulka.

Another author strongly critical of logotherapy was Edith Weisskopf-Joelson, who explicitly accused logotherapy of being “not a “scientific” psychotherapeutic school in the traditional sense but that it is, instead, a philosophy of life, a system of values, a secular religion” and that stated that “many contemporary psychotherapists find it difficult to accept the philosophical or religious aspects of Logotherapy and of psychotherapy in general” (Weisskopf-Joelson, 1975, 238). According to this author, the central logotherapeutic concept, that of the meaning in life, plays a psycho-hygienic role “which greatly resembles the function of God in the Judeo-Christian world view” and when a person discovers her/his meaning in life, it works as a guide that determine her/his actions “in the same manner as the awareness of a deity guides the actions of the religious Christian or Jew” (Weisskopf-Joelson, 1975, 238). It is evident that Weisskopf-Joelson considers logotherapy as a secular religion. Ultimately, taking this author's ideas to their extreme, logotherapy would be the substitute for religion in a secularized world. This is evident in the following Weisskopf-Joelson’s assertion: “by proposing a secular equivalent to the concept “God,” Logotherapy reveals itself as being a philosophy of life, or a faith, more than a scientific therapeutic school” (Weisskopf-Joelson, 1975, 238). The religious nature of logotherapy, in Weisskopf-Joelson’s opinion, would also be revealed in its proposal of values, in the conception of life similar to religious ideas about immortality, and in the way Frankl would have spread his ideas. Not only logotherapy deserves such criticism, but the person of Frankl himself, which shows in the final paragraph of his work (Weisskopf-Joelson, 1975):

I wish to say that I have always perceived Frankl as a mixture of prophet, guru and preacher disguised as a psychiatrist who disseminates his message in a language to which men and women of the twentieth century are likely to listen, the language of psychology. But the world, and perhaps the man himself, has taken the disguise too seriously and has become oblivious to the prophetic person who stands behind the psychiatric cloak. (240)

Our opinion is that logotherapy does not have a religious bias because Frankl stresses that religiosity can only be considered a psychotherapeutic resource if the patient himself raises it in psychotherapy, and that religion can never be considered an imposition by the therapist because this would be a disrespectful invasion of the patient’s convictions and beliefs.

However, one thing is what Frankl thinks about the role of religion in general and about the personal religiosity of the therapist and the patient in particular, and another is the possible religious impregnation, so to speak, of logotherapy. In this regard, it is interesting to note that Morgan (1983), one of the main disseminators of logotherapy in the USA, pointed out the religious component, specifically Jewish, in Frankl’s psychotherapeutic proposals, whose references, however, are not made explicit by Frankl (Morgan, 1983):

The surprising feature of Frankl’s psychotherapeutic formulations is that throughout he consistently makes inferential comments about the religious dynamic operative in his theory while constantly omitting any specific reference to its fundamentally Jewish character. Especially, he consistently fails to refer, even at the most commodius opportunities, to the presence of a strong element of Hassidic teachings (…) we cannot be far wrong in the identification of major Jewish principles in Viktor Frankl’s psychology (…) unquestionably used the philosophical teachings of the Hassidic rabbis in his considerations of life’s meaning. (pp. 186-187).

Before Morgan, as he himself pointed out, other authors (e.g., Rubenstein, 1968) affirmed that logotherapy and the will to meaning were no different from the rabbinic effort to achieve an ordered and meaningful universe. It also refers to the thought of the philosopher and rabbi Abraham Joshua Heschel (1907–1972), in whose rabbinic teachings one can find elements that absolutely coincide with the essentials of logotherapy (e.g., Heschel, 1968), particularly those related to the transcendent, that is, not merely subjective, character of meaning in life.

Such coincidences could be explained by the fact that logotherapy has a Jewish metaphysical background. Therefore, the fact that Frankl’s practiced Judaism could have essentially influenced his theory on meaning in life. Morgan (1983) pointed out, without claiming to attribute to Frankl a conscious rabbinic bias, that logotherapy, in addition to being an existential psychology, is a rabbinic philosophy or, at least, essentially rests on it, which is precisely what gives it strength.

Rabbinic metaphysical elements are perhaps particularly evident in the explicit references to transcendence and religion in Frankl’s work. His qualifying work as a doctor of philosophy, *Der Unbewusste Gott* (Frankl, 1948, 2002), and *Gottsuche und Sinnfrage: Ein Gespräch*, the book co-written with Lapide (Frankl & Lapide, 2005), are noteworthy in this respect.

It seems that it cannot be denied, despite Frankl’s attempts to distance logotherapy from religion after May’s criticism in the 1960s, that his theory is strongly influenced by religion, specifically his Judaism and the Hasidic tradition in a city like Vienna, where the Jewish community was strongly tied to its religious beliefs and traditions; and in a family, such as Frankl’s, which was religiously observant and linked to the Rabbi Judah Loew ben Bezalel (c. 1520–1609), both in its metaphysical and anthropological foundations and in its relation to the motivational theory of the meaning of life and psychotherapeutic practice.

However, in spite of the importance of the spiritual in the sense of the noogenic as ontologically superior to the psychophysical and all that this implies, that is, the relationship with values, the role of conscience, etc., and of the specifically religious in the existential analysis of the constitutive religiosity of the human being, unconscious religiosity, the relationship between the meaning of life and religion, the role of God in the moral conscience, etc., Frankl did not distinguish a specifically religious dimension in the human ontological structure. This led Shea (1975) to ask:

Is his description of the religious man in any sense part of an essential phenomenology, and if so, why is the religious dimension not a part of his ontology? But if his description of the religious man is simply a description of the values that some individuals have found in their lives, on what grounds is Frankl so partial to these values? And what is the thrust, in terms of his existential analysis, of references such as “an entity higher than ourselves”? (181-182)

In addition, Shea points out that, according to Frankl, the question of God is posed in terms of ultimate meaning and supermeaning, “which supercedes “dimensionally” man’s capacity as a finite being and yet is capable of being grasped in an existential act” (Shea, 1975, 182). It is a supermeaning that surpasses the human capacity of apprehension and rational understanding, and it is only accessible through personal commitment that would emerge from the deepest part of the person and be rooted in his/her total existence: it would be an existential act that could be described as basic trust in Being. The ultimate meaning is confronted as an option to the «ultimate absurdity» of life, so that the person is faced with a choice between the two, with his or her choice being a matter of faith that would reveal personal trust in God. Frankl’s approach would imply the belief that “all men, at least unconsciously, are basically religious, and that trust in ultimate meaning and faith in being, however, dormant, are indispensable for existence [that is]; there is, then, in all men a religiosity which is easily repressed” (Shea, 1975, 183).

On the question of the religious dimension in man in Frankl’s existential analysis, there are three basic aspects, namely: (1) that it can only be considered personally through an act of existential affirmation, which some perform and some do not, (2) that every individual is inherently religious, but, for a variety of reasons, some have their religiosity repressed or unconscious, and (3) that Frankl himself is a man of faith who trusts in the ultimate Self (Shea, 1975, 183). The dimensional ontology of logotherapy is ambiguous in relation to religion because, although it admits the spiritual dimension of the person and his/her openness to transcendence, it does not include it as a specific ontological dimension (Shea, 1975).

## Religion as Spirituality in Logotherapy, not (Necessarily) as a Religious Creed

Reitinger (2015, 353) stated that, according to Frankl, “since we are always in relation with God, it is possible to orient ourselves toward meaning and to answer our life questions in a meaningful way. By realizing meaning, the person draws closer to God.” However, by spirituality, Frankl did not mean this or that formal religion, but rather the human tendency to discover, wonder, and contemplate the “mystery” of life, the grandeur of nature, and the like. According to Wong (2002), Frankl clearly distinguished between spirit, spirituality, and religion:

Spirit refers to one of the dimensions of humanity. Spirituality is manifest in a person’s quest for meaning. Religion encompasses the ultimate meaning, super meaning, as well as God. He clearly recognizes the importance of religion but is reluctant to be considered religious. He equates authentic religion with deep spirituality. (108)

Morgan (2013) noted:

Neither a proponent nor an opponent of a faith-based worldview per se, Frankl simply intends for spirituality not to be tied up with a specific notion of religion. When faith helps a person through the day, Frankl has no objection to it. When religious worldview and ethos stifle, cripple, or delude an individual, Frankl is opposed to it. What Frankl means by “spirituality” as a fundamental component of human nature is man’s capacity for a sense of awe, wonder, and mystery, even reverence, in assessing the meaning, value, and purpose of one’s own personal life. The surprising feature of Frankl’s psychotherapeutic formulations is that, throughout them, he consistently makes inferential comments about the operative religious dynamic in his own theory while constantly omitting any specific reference to its fundamentally Jewish character (Frankl, 1961). The connectedness of all things, as experienced in moments of high sensitivity or even ecstasy, is the role spirituality plays in the human character. A deeply felt sense of beauty, power, and wonder in the universe, a heightened experience of integrality, what I have elsewhere chosen to call “systemic integrality,” constitutes what spirituality means in logotherapy (Morgan, 2009). (…) Whether one is a theist, an atheist, or an agnostic, Frankl contends that the dynamics of spirituality can equally and meaningfully operate within a person’s life, bringing value and purpose. (325)

In Péter’s (2008) opinion, logotherapy:

[…] is not connected directly to any form of confession. At the same time, it can be regarded as partly religious because the theory and practice of logotherapy does not stop at the level of existential psychotherapy, namely, the humanistic turn, but continues the questioning. (168)

Certainly, logotherapy includes, as an essential element, the reference to transcendence in a purely religious sense: “The image of the human being cannot be accomplished within the limits of immanence. The human being perceives itself as the image of God –or else becomes its own distorted image” (Cfr, 230; as cited in Péter, 2008, 168).

As noted above, Grollman (1965) stated that Frankl’s writings were interlaced with quotations from authoritative Jewish sources and that his works breathe the spirit of Judaism. Although in his opinion “Frankl believes that religion is more important than many psychotherapists have admitted and more relevant to the patient than the paucity of physicians would indicate” (Grollman, 1965, 35), logotherapy is not a religion, but rather it “is implicitly religious in the sense that it assists in aspiring for the summum bonum. It is not explicitly religious as a presentation of sectarian religious dogma” (Grollman, 1965, 35).

## Conclusion

Like many psychologists throughout the twentieth century, Frankl was interested in religion, its place in human nature, and its relationship with psychotherapy. Without a doubt, religion had an important weight in his person and thought, and it occupied a significant place in logotherapy, in both its anthropological and therapeutic dimensions. Frankl did not avoid the religious theme when dealing with the nature of the human being, the relationship between religion and psychology, and the potential role of faith in psychotherapy.

Thus, Frankl considered that human beings are naturally religious (sharing this idea with the Fathers of the Church and scholasticism), that there is a spiritual unconscious (in addition to a pulsional unconscious, as defended by Freud, with the former being more important than the latter in the dynamics of significant human behavior), that the therapist should be open to the patient's religious approaches as a psychotherapeutic resource (when the patient exposes them during the intervention), and that religion can have a psycho-hygienic effect on mental health. Certainly, for Frankl, religion occupies (or can occupy, if it is consciously experienced) a central place in people’s lives.

Criticisms that logotherapy is a kind of religion disguised as psychotherapy, or a religious psychotherapy, or some kind of profane religion are neither adequate nor fair. Frankl always defended the differences and limits between religion and psychotherapy, between the priestly cure of souls and the medical cure of souls, between the salvific objective of religion and the hygienic objective of psychotherapy. In our opinion, some critical authors have failed to appreciate Frankl’s effort to expose this distinction with complete personal and intellectual honesty. Other authors, such as Péters (2008) and Morgan (2013), were much more cautious in their assessments of this issue.

Possibly, such criticisms were due more to reticence about, if not rejection of, religion by the critics. It should be kept in mind that psychology had for centuries been subject to metaphysics, particularly to a religious and specifically Christian metaphysics. For some, to admit, even to a small extent that religion and personal faith could have a place in psychology and psychotherapy was a mistake and did a disservice to the scientificity of the former. Hence, Frankl, who not only was not critical of religion, but unambiguously admitted it as a central dimension of human nature and considered its potential psychotherapeutic role, was viewed with suspicion, especially when his conception of religion was Jewish and very close to Christianity. It is remarkable the influence that Frankl's thought has had and continues to have among Catholics, for example. Regarding to that, Pytell (2015) stated:

[…] all of them [some Frankl’s Christian disciples] were interested in using Frankl’s logotherapy to buttress Christianity [and] the uncritical praise reflected the solace Frankl’s logotherapy provided for the religiously oriented. To them, Frankl probably seemed like a breath of fresh air—here was a therapy that could be used to promote a Christian world view.

[…]

Frankl’s psychology found little resonance among religiously oriented Jews. Apparently mainly Christian thinkers were drawn to study with him, and his ideas about guilt, death, and suffering were very compatible with a Christian world view. (163)

With regard to the relationship that Judism and Christianity might have with logotherapy, Frankl stated the following in a footnote to his book *The Doctor and the Soul* (Frankl, 1986):

The unique achievement of Mosaic monotheism may well consist in its conveying to the human race the permanent consciousness of a divine authority. Man is seen as a being standing before God, thereby intensifying man’s consciousness of responsibility by presenting his life task to him as an assignment from the Divine. But we must not forget that the moral urge springing from this view was chiefly concerned with what we have called creative values. It must therefore appear all the more remarkable to us when we realize that Cristianity has placed in the foreground of man’s moral consciousness the kind of values we have called attitudinal—the third of the three main categories of possible values. For the Christian existence, taken in the perspective of the cross, of the Crucified One, becomes ultimately and essentialy a freely chosen imitation of Christ, a “passion”. It remained for Protestantism to install the further element; by emphasizing the concept of grace, Protestantism deepened man’s sense of responsibility in regard to the second category of values, experiential values. For in terms of the idea of grace, which is so cardinal a point in Protestant theology, all of man’s encounters with valuational experiences constitute receiving a gift of God (grace). All this, it seems to us, suggests a coherent relationship between the three categories of values on the one hand and the three principal branches of Occidental religion on the other. (60)

Frankl’s position about human religiosity and the relationship between religion and psychotherapy is correct and valuable, and it should be judged flexibly and without suspicion. Admitting that man is naturaliter religiosus, that his life can be strongly (and positively) influenced by faith, and that psychotherapy can benefit from it should not be problematic for a mature science, as psychology undoubtedly is today and as so many empirical studies show (e.g., Marques et al., 2022; Spencer et al., 2016; Sweet & Paul, 2022).

## Footnotes

1 Author’s translation from Spanish edition.

2 Author’s translation from a Spanish edition.
